# Underwater Robots and Key Technologies for Operation Control

**DOI:** 10.34133/cbsystems.0089

**Published:** 2024-03-27

**Authors:** Linxiang Sun, Yu Wang, Xiaolong Hui, Xibo Ma, Xuejian Bai, Min Tan

**Affiliations:** ^1^ School of Automation, Harbin University of Science and Technology, Harbin, China.; ^2^ State Key Laboratory of Multimodal Artificial Intelligence Systems, Institute of Automation, Chinese Academy of Sciences, Beijing, China.; ^3^ School of Artificial Intelligence, University of Chinese Academy of Sciences, Beijing 100049, China.; ^4^ School of Electrical Engineering, Liaoning University of Technology, Jinzhou, China.; ^5^ Institute of Automation, Chinese Academy of Sciences, Beijing, China.

## Abstract

Over time, the utilization of the Underwater Vehicle-Manipulator System (UVMS) has steadily increased in exploring and harnessing marine resources. However, the underwater environment poses big challenges for controlling, navigating, and communicating with UVMS. These challenges have not only spurred the continuous advancement of related technologies, but also made the development of the UVMS even more captivating. This article firstly provides a review of development status of the UVMS and discusses the current limitations and future directions, and then reviews in detail the dynamic and hydrodynamic modeling methods, and analyzes the principles, advantages, and disadvantages of various approaches. Then, we try to review 2 key technologies of operation control methods, including underwater positioning and navigation technologies and vehicle-manipulator coordinated control approaches. Finally, a reasonable prospect for the future development of UVMS is given.

## Introduction

With the rapid development of society and economy in recent decades, problems such as shortage of terrestrial resources and environmental degradation have become increasingly prominent [1]. The vast ocean is not only the birthplace of life, but also a huge treasure house of resources [2]. The ocean is our future. Therefore, marine science and technology, especially marine high technology, has become one of the most dynamic and promising scientific/technological fields in the 21st century. In recent years, bathyscaphe, underwater robots, and advanced underwater equipment have gradually become hot topics in the development of marine high technology industries [3]. The Underwater Vehicle-Manipulator System (UVMS) plays a vital role in the development and utilization of marine resources.

At present, most of the UVMS that have been industrialized are Remotely Operated Vehicles (ROVs). The ROV can establish connections with the mother ship using an umbilical cable that can not only supply power and transmit operation instructions, but also obtain information such as images returned by the ROV [4]. Nevertheless, the ROV has many limitations in terms of range, cost, automation, situational awareness, decision-making, and susceptibility to damage. Hence, to address the various limitations of the ROV, developing the UVMS with high autonomous operation capability and high intelligence has become the focus of current and future research, and these advanced vehicles offer significant potential for safer, more efficient, and cost-effective underwater operations in various domains like scientific research, resource exploration, infrastructure inspection, and marine conservation.

In the past 10 years, some scholars have tried to review related works in this field. Wang et al. [4] and He et al. [5] reviewed the development status of autonomous underwater vehicles (AUVs). Kumar et al. [6] systematically reviewed control methods, and discussed simulation and modeling approaches of underwater robots. Salazar et al. [7] and Panda et al. [8] reviewed different hydrodynamic modeling methods.

Significant progress has been made, particularly in terms of modeling and underwater operation control. However, there are still problems and challenges: (a) accurate modeling and hydrodynamic coupling problem of the UVMS, (b) underwater autonomous positioning and navigation, (c) precise motion control of the UVMS, and (d) vehicle-manipulator coordinate control, among others.

This review provides a comprehensive summary and outlook on the current development status and key technologies of UVMS.The remainder of this paper is organized as follows. The “Development Status of the UVMS” section reviews the development and research status of the UVMS. The “Key Technologies of the UVMS” section details the dynamic and hydrodynamic modeling methods and the autonomous operation control methods. The “Discussion” section discusses the existing issues and future development directions. Finally, the “Conclusion” section presents the conclusion.

## Development Status of the UVMS

The earliest human exploration of underwater robots can be traced back to the wooden spherical submersible manufactured by the Italian Tarcilia in 1554, which has a history of more than 400 years [4]. The development history of underwater robots is very long.

There are many types of UVMS, which can be classified according to different characteristics. According to weight and size, the UVMS can be divided into micro-miniature type, small type, lightweight industrial grade, industrial grade [9], etc. According to the operation mode, the UVMS can be divided into remote-control type, semi-autonomous type (hybrid type), and autonomous type. According to the propulsion method, the UVMS can be divided into propeller propulsion type and bionic propulsion type [10].

In the 1960s and 1970s, the ROV experienced a period of vigorous development and gradually evolved into a well-established industry in the next decades. The AUVs started to emerge in the 1990s. In recent decades, with the continuous advancement of technology and ongoing upgrades to both software and hardware, the AUV has made significant progress. However, it is important to note that some technologies still face bottlenecks. Therefore, the current underwater robots are mainly semi-autonomous types. The advantage of this hybrid type is that the robot body and the manipulator not only can be remotely operated by operators, but also can autonomously complete some specific tasks in specific scenes.

The United States started early in this field and has achieved a high level of research and development. In the late 1950s and early 1960s, the Naval Ordnance Station Louisville (NOSL) began to develop the UVMS, and finally successfully developed the first-generation CURV-I in 1960. Since then, on the basis of CURV-I, the NOSL had further developed the second and third generation, CURV-II and CURV-III [11], respectively. Up to now, the latest one, CURV-21 [12], could carry out salvage and rescue tasks at the depth of 6,000 m. The CURV-21 is equipped with high-power thrusters, a pair of 7-degree-of-freedom (Dof) manipulators, HD cameras, and an active sonar. In March 2023, the U.S. Navy successfully salvaged the F35 fighter that sank in the South China Sea using the CURV-21.

The Woods Hole Oceanographic Institution (WHOI) successfully developed the JASON [13,14] in 1988. The maximum operation depth can reach 6,500 m. In 1995, WHOI successfully developed a semi-autonomous double-buoy UVMS named NEREUS [15]. The NEREUS is equipped with a 6-Dof hydraulically driven manipulator, and its maximum diving depth can reach 10,902 m. On the one hand, NEREUS can work in autonomous mode for seabed topographic mapping, photography, sampling, and other tasks. On the other hand, NEREUS can establish the real-time communication with the mother ship through the optical fiber to complete the target object grasping task. In 2014, on the basis of NEREUS, WHOI began to develop Nereid-UI [16] for polar exploration.

In 1991, the Autonomous Systems Laboratory of Hawaii University successfully developed the Omni-Directional Intelligent Navigator, an experimental spherical underwater robot [17–19]. Subsequently, the laboratory developed the semi-autonomous underwater robot SAUVIM (Semi-Autonomous Vehicle for Intervention Missions) [20] for intervention tasks. SAUVIM is powered by a nickel-metal hydride battery pack and equipped with a 7-Dof manipulator [21], and can autonomously achieve navigation, positioning, and target fishing and recovery operations [22,23] in the marine environment.

In 2016, the Robotics Laboratory of Stanford University successfully developed a humanoid semi-autonomous underwater robot, Ocean One [24]. It weighs only 200 kg, and is equipped with a pair of highly precise, high-torque and 7-Dof humanoid manipulators. This pair of manipulators can be used to interact with surroundings with human-level dexterity [25]. Thanks to the 24 Dof, the Ocean One is very suitable for the target objects salvaging in the middle and shallow seas [26]. The Ocean One’s accomplishments mark the beginning of a new level of man–machine cooperation.

Generally, dual manipulators work more efficiently. At the same time, the streamlined shape makes the robot reach the working area faster. Combining the above 2 characteristics, in 2018, the Houston Mechatronics company in the U.S. developed Aquanaut, a deformable dual manipulators UVMS [27]. The Aquanaut can work in places with water depths exceeding 3,000 m. The Aquanaut can reach the vicinity of the working area in the flatfish-like form, and then gradually expand into the work form.

In addition, Japan and South Korea have been researching and developing the UVMS for many years. The representative ones like the Twin-Burger [28] with double-buoy structure was developed by the Institute of Industrial Science of Tokyo University in 1992; the full-sea depth UVMS KAIKO [29,30] was developed by Japan Agency for Marine-Earth Science and Technology (JAMSTEC) in 2004. On the basis of KAIKO, JAMSTEC developed the 3,000-m-level HYPER-DOLPHIN [31], the 7,000-m-level UROV7K, and the latest one, KAIKO MK-IV [32]. The KAIKO MK-IV is equipped with conductivity–temperature–depth (CTD) sensors, various types of cameras, navigation equipment, and a pair of 7-Dof manipulators. In 2016, the Kyushu Institute of Technology developed the 2,000-m-level Tuna-Sand [33]. The Tuna-Sand only weights 380 kg and is equipped with 8 propellers and a 4-Dof manipulator. With the help of the visual tracking systems, the Tuna-Sand can complete biometric identification and sampling tasks on the seabed [34–36], which helps to understand resource diversity for sustainable use of marine resources.

In 2017, the Korea Research Institute of Ship and Ocean Engineering carried out the autonomous intervention operation project and developed the UVMS Korea Research Institute of Ship and Ocean Engineering (KRISO) [37]. The KRISO is equipped with 8 propeller thrusters, a 7-function manipulator, and sensors such as Doppler Velocity Log (DVL) and Inertial Measurement Unit (IMU). The KRISO can carry out various underwater sensing and intervention operations.

European countries are also keen on ocean exploration and the development of marine resources. The National Oceanography Center (NOC) of the United Kingdom has developed the industrial-grade deep-water UVMS Isis [9]. The Isis weighs 4,000 kg when fully loaded in air, with dimensions of 2 m × 2 m × 2.5 m. It is equipped with HD cameras, a flexible manipulator, and a hydraulic power pack that enables hydraulic functions such as cable cutter, tool drawer, etc. The Isis is capable of conducting sampling, salvaging targets, and drilling seabed sediments at a depth of up to 6,500 m. The Saab Seaeye company from the UK released the industrial-grade UVMS Partner-XT in 2019 and Cougar-XT in 2021, respectively. They are equipped with high-power propellers capable of withstanding strong currents, as well as a pair of manipulators that can work together to complete tasks such as dam maintenance and underwater salvage.

In 2017, the Italian Institute of Automation and Intelligent Systems developed e-URoPe [38]. This vehicle can be freely equipped with various types of sensors and loads to complete different tasks such as object sampling, underwater manipulation, collaboration with underwater operators or other vehicles, and underwater terrain mapping, among others. A team from the German Research Center for Artificial Intelligence developed the dual-arm intervention UVMS Cuttfish in 2022 [39]. The Cuttfish has 8 propellers and can support both horizontal and vertical working modes,and its 2 manipulators on the abdomen can be folded and stored.

The European Union (EU) initiated the TRIDENT EU FP7 project [40] with a primary focus on advancing underwater autonomous operation technologies, aimed at furthering the research in the UVMS. The member states that participate in the project jointly developed a set of robot systems with autonomous perception and motion planning capabilities. The systems mainly include 3 functional modules: (a) the GINORA-500 AUV (robot body) [41] developed by the University of Girona, (b) a 7-Dof manipulator designed by Graal Tech, and (c) the end effector and the multi-functional dexterous hand system developed by the University of Bologna. After years of adjustment and experimentation, the system currently has a high ability to operate autonomously. Through the coordinated control of the body and the manipulator, coupled with the autonomous intervention technologies, the system can automatically complete target searching and approaching, valve salvaging or switching, platform return, and a series of autonomous operation tasks in the pool environment.

In 2017, the EU developed the DexRov [42]. The DexRov can establish the connection with the base station using exoskeleton virtualization technology, and is equipped with a 6-Dof manipulator and a 3-Dof end effector, which employs advanced artificial virtual technology to achieve precise control [43]. The DexRov will be mainly used for offshore observation and equipment maintenance in the future.

The research and development of the UVMS in China is relatively late, but with the increasing demand for ocean development, China has also made great progress. In 2018, Shanghai Jiaotong University independently developed the 6,000-m-class UVMS Hailong-11000 [44]. The Hailong-11000 is equipped with a variety of advanced instruments and equipment. Up to now, a series of experiments have been successfully carried out in the northwest pacific. Since 2005, the Shenyang Institute of Automation, Chinese Academy of Sciences had started to develop a variety of underwater vehicles, one of which was a semi-autonomous one named Arctic. The Arctic followed the Chinese Arctic Scientific Expedition team and participated in monitoring missions of the marine environment under the Arctic ice. The Arctic successfully obtained a large amount of marine environment data and could provide data support for further Arctic scientific expedition missions [45].

As technology continues to advance, research centers and major enterprises gradually tend to research and develop the bionic-UVMS [46]. In 1995, Triantafyllou and Triantafyllou [47] successfully developed the world’s first bionic-propulsion underwater robot RoboTuna and carried out research on the theoretical method of efficient caudal fin propulsion. In order to adapt to the narrow and complex underwater operation environment and ensure the high-efficiency completion of underwater operation tasks, Norway’s Eelume AS company developed the snake-shaped UVMS Eelume [48]. The main body of the Eelume is composed of multiple modular joints, and each joint has 2 Dof, so the number of joints can be flexibly configured according to the type of task. The Institute of Automation of the Chinese Academy of Science (CASIA) has developed the RobCutt-I [49], which was propelled by the undulatory fin. The undulatory fin has the characteristic of low-speed stability. Therefore, it is very suitable for hovering operations in still water environment. On the basis of the RobCutt-I, the CASIA has successively developed RobCutt-II [50] and the latest one, RobCutt-III [51]. The RobCutt-III is equipped with the flexible undulatory fins and a more stable inverted triangular structure.

**Fig. 1.**
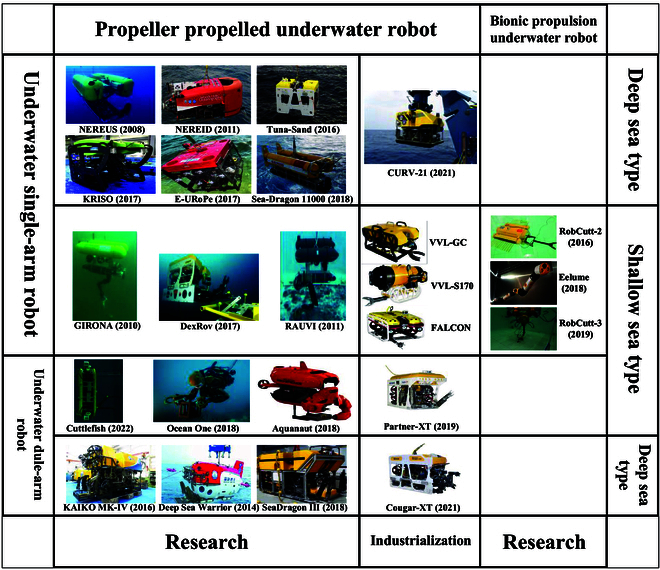
Classification and summary of the UVMS.

Based on the above research status, the UVMS is summarized according to different dimensions, as shown in Fig. 1. It is evident from Fig. 1 that significant progress has been achieved. Different test prototypes have been developed according to different applications, and some of them have also achieved industrialization. As research into high-efficiency bionic propulsion theory deepens, the bionic propulsion UVMS has garnered increasing attention, leading to the development of experimental prototypes. However, most of the UVMS currently work in ROV mode. There is a need for further improvement in the autonomous operation capability.

In summary, most UVMS lack the ability to operate independently and autonomously, the promotion mode is relatively simple, and the level of industrialization is limited.

## Key Technologies of the UVMS

**Fig. 2.**
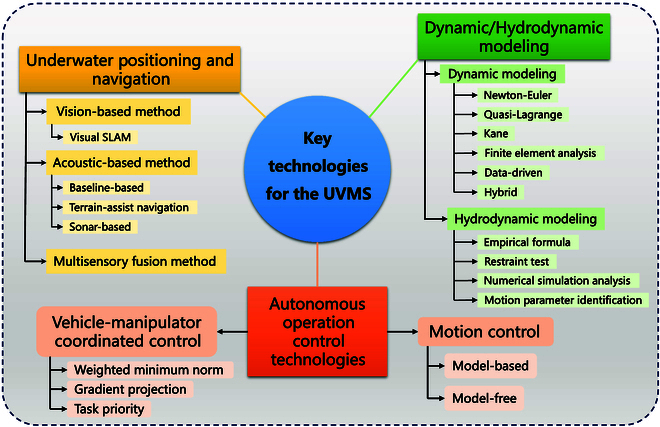
Overview of the reviewed key technologies.

As one of the most critical technical issues in the research, underwater operations of the UVMS have always been the focus. To meet the needs of autonomy, the motion characteristics and control characteristics of the UVMS are relatively special. It is necessary to not only achieve stable control but also ensure control accuracy and efficiency. The research directions of the UVMS control methods mainly focus on the following aspects: dynamic/hydrodynamic modeling, underwater positioning and navigation, motion control and coordinate control, etc. The overview of key technologies related to the UVMS reviewed in this article is shown in Fig. 2. Due to the complexity of and unfamiliarity to the operating environment, how to formulate control strategies has always been a key and difficult point in the research. The main reasons why it is difficult to design control strategies are as follow:

1. The UVMS system is highly coupled, nonlinear, and time-varying.

2. It is difficult to obtain real-time and accurate hydrodynamic coefficients when UVMS is moving.

3. The actual motion has a great correlation with the density and viscosity of the fluid.

4. Large sea current disturbance.

5. The shift of overall center of gravity and buoyancy caused by different loads.

### Dynamic modeling of the UVMS

The dynamic model serves as the foundation for structural optimization, simulation, and controller design of the robots. For the UVMS dynamic modeling, it is essential to consider not only the models of the robot body and manipulator but also the coupling relationship between them, as well as the hydrodynamic model [52,53]. To the best of our knowledge, after decades of development, dynamic modeling mainly uses the following methods:

The Newton–Euler (NE) method [54] is based on the Newton’s Second Law of Motion and Euler’s First and Second Law of Motion. It is the most commonly used dynamic modeling method. The dynamic and kinematic states are recursively transferred to either the base of the robot body or the end of the underwater manipulator. Finally, establish the model of the force and motion state of the robot;

The Quasi-Lagrange (QL) method [55] is based on the Lagrange equation. A generalized recursive relationship between forces, torques and the kinetic, potential energy of the system could be established. By using the Kirchhoff transformation matrix, the Lagrange dynamic model in the satellite coordinate could finally be obtained.

The Kane method [56] is based on the Kane equation, as shown in Eq. 1.

$$F_{r}+F_{r}^{*}=0\left(r=1,\ldots ,N\right)$$ (1)

where *F*_1_, …, *F_N_* denotes the generalized active forces of the robot system, and $F_{1}^{*},\ldots ,F_{N}^{*}$ is the generalized inertia forces associated with the robot system [57]. The forces in each part of the system are projected on generalized speeds. This method effectively reduces the number of differential equations, so it can be used for complex system modeling.

The Finite Element Analysis (FEA) method [58] is a numerical method. By establishing a discrete mathematical model of the UVMS and applying appropriate boundary conditions, the numerical dynamic model of the UVMS could be solved. Not only the coupling relationship between the robot body and the manipulator, but also the deformation of the flexible manipulator could be obtained.

The Data-driven method [59] is based on Neural Networks. The dynamic model of the UVMS is regarded as a black box. The continuous residuals caused by the environmental uncertainty could be compensated by the network learning. This method has strong environmental adaptability and robustness.

The Hybrid method [60,61] combines the traditional physical methods and the data-driven methods, and not only has theoretical interpretability and application generalization but also has strong environmental adaptability.

Table 1 presents the summary of the related dynamic modeling methods.

**Table 1.** Summary of the dynamic modeling methods reviewed

Method	Advantage	Limitation
NE [54,131]	1. Intuitive expression form	Difficult to remove constraints and establish closed-form kinetic equations
	2. High computational efficiency	
	3. Real-time force and torque data	
QL [55]	1. Easy to incorporate new subsystems	Difficult to handle some complex algebraic and differential operation
	2. Easy manipulator feedback handling	
	3. Function of the system control input	
Kane [132]	1. High modeling efficiency	Calculating joint inertia is challenging
	2. Few differential equations	
	3. External force intuitively introduced	
FEA [58]	1. Wide applicability	Difficult to remove discretization error
	2. Flexible dynamic modeling	
Data-driven [133]	Strong environment adaptability and robustness	1. Data quality limits modeling
		2. Limited generalization ability
Hybrid [60,61]	1. Theoretical interpretability	1. Sensitive to hybrid rules
	2. Strong generalization	2. Poor scalability
	3. Strong environment adaptability	

NE, Newton–Euler; QL, Quasi-Lagrange; FEA, Finite Element Analysis.

Furthermore, the UVMS is also affected by the surrounding water flow, a phenomenon referred to as the hydrodynamic effect, which can directly affect the accuracy and stability of the underwater operation. Therefore, it is necessary to establish hydrodynamic models. It is generally accepted that the hydrodynamics can be described as polynomial forms composed of hydrodynamic coefficients and motion parameters that related to the robot and the surrounding fluid characteristics. However, due to the strong nonlinearity and uncertainty of the hydrodynamic coefficients, there is currently no unified and effective methods to solve [62]. The current methods for obtaining hydrodynamic coefficients mainly include the Empirical Formula (EF) method, the Restraint Test (RT) method, the Numerical Simulation Analysis (NSA) method, and the Motion Parameter Identification (MPI) method. Table 2 presents the summary of the related hydrodynamic modeling methods. To further improve the quality of modeling, hybrid methods have progressively emerged. In 2022, Lin and Chiu [63] proposed a method based on towing test and numerical simulation, and the results could be used to generate a comprehensive database of hydrodynamic coefficients for underwater robots.

**Table 2.** Summary of the reviewed hydrodynamic modeling methods

Method	Model generalization	Advantage	Limitation
EF [64]	Bad	Simple and fast	Invalid for complex models
RT [65]	Bad	High-quality data available	Complex data interpretation
NSA [66,67]	Good	1. High flexibility	Model uncertainties
2. Cost-effective			
MPI [68,69]	Good	1. High model reliability	1. Difficult to collect valid data
2. Learning ability		2. Risk of over-fitting

EF, Empirical Formula; RT, Restraint Test; NSA, Numerical Simulation Analysis; MPI, Motion Parameter Identification.

The EF method is for simple-shaped underwater robots [64]. The relatively reliable hydrodynamic coefficients can be directly and efficiently obtained according to the previous EF. However, for those underwater robots with complex shapes, this method cannot meet the accuracy modeling requirements. This method has been gradually phased out with the development of modern computer technology.

The RT method uses a scaled-down model to test in the pool, collect relevant data, and finally convert the hydrodynamic coefficients of the entity according to the proportional relationship [65]. High accurate hydrodynamic coefficients can be obtained through this method, but it requires a suitable test site and advanced measuring instruments. It has a high threshold for use.

The NSA method [66,67] obtains the corresponding hydrodynamic coefficients by establishing the network model of the underwater robots and performing numerical simulation through Computational Fluid Dynamic (CFD) software such as the FLUENT, CFX, STARCCM+. Not only the inertial hydrodynamics but also the viscous hydrodynamic coefficients among the model can be calculated via regression analysis.

The MPI method [68,69] is similar to machine learning. It combines the physical and numerical methods. On the basis of the pre-built hydrodynamic model and a large number of motion data of the underwater robots, using optimization algorithm such as the Genetic Algorithm (GA), the unknown hydrodynamic coefficients can be identified.

In summary, the dynamic modeling methods are relatively mature. However, there is still a lack of simple and effective methods for hydrodynamic modeling. Furthermore, while there are numerous studies on the dynamic modeling of propellers, there is a lack of research on the dynamic modeling of the bionic propulsion mechanisms.

### Underwater positioning and navigation

Underwater environments are highly unstructured and non-linear, full of various noise. All of these factors have brought great challenges to the positioning and navigation of the UVMS. Currently, the underwater navigation consists primarily of the vision-based methods [70–72] and the acoustic-based methods [73–75], as well as the multisensory-fusion methods [76–78], which has developed gradually over the past few years.

#### Vision-based underwater positioning and navigation methods

The vision-based methods are based on the machine vision and image processing technologies. These methods are capable of stable long-term operation without accumulating errors over time [79]. The commonly utilized vision-based technology in the underwater environment is visual Simultaneous Location and Mapping (SLAM) [80]. The basic steps of SLAM are as follows: (a) image acquisition, (b) front-end image processing, (c) back-end optimization, (d) loop closure, and (e) locating and mapping. SLAM methods can provide reliable localization for underwater robots in unknown environments, and construct the real-time map of the surrounding environment.

In 2018, Hidalgo et al. [81] verified the possibility of applying the ORB-SLAM [82] underwater, and evaluated the effects in different scenarios. The results have shown that the light degradation and turbidity affected the quality of images and number of feature points, and indirectly affected the positioning accuracy. Aiming at the blurred problem of underwater images, Chen et al. [83] proposed an image enhancement method based on Generative Adversarial Network (GAN) and deployed it on the ORB-SLAM framework. The experiments have proved that the performance is considerably improved using the proposed solution. At present, the underwater SLAM methods can be divided into monocular vision based and stereo vision based.

The monocular vision-based methods have a simple structure, are low cost, and can be utilized indoors and outdoors. In 2016, Hong et al. [84] proposed a robust SLAM method with the Augmented State Kalman Filter (ASKF) and provided an idea to address the problem of SLAM in the unstructured underwater environment. The proposed approach can effectively improve the performance of navigation and map construction. In 2018, Burguera and Bonin-Font [85] proposed a multi-session monocular approach that adopts the local single-session, global multi-session loop detectors and the optimizer based on the Iterated Extension Kalman Filter (I-EKF). The experiments have shown that it could not only achieve fast matching, but also reduce the computational effort without reducing the positioning accuracy. Wu and Gao [86] proposed an improved VINS-MONO [87] algorithm, which used a Features from Accelerated Segment Test (FAST) feature point extraction approach and an Inverse Optical Flow (IOF) algorithm to improve the extraction speed and accuracy, respectively, and adopt a Global Bundle Adjustment (GBA) optimizer. Westman and Kaess [88] proposed an algorithm based on the Pose Graph Bundle Adjustment (PGBA). Hong and Kim [89] addressed a method for 3D reconstruction. This method introduces the loop-closure Factor Graph Bundle Adjustment (FGBA) into the SLAM system, to achieve precise trajectory estimation and reconstruction. Silveira et al. [90] proposed a bio-inspired SLAM named DolphinSLAM, which was an extension of RatSLAM [91]. It can work well with low-resolution visual image data and use Continuous Attractor Neural Network (CANN) as optimizer. Table 3 summarizes the underwater monocular visual SLAM methods.

**Table 3.** Summary of the reviewed underwater monocular visual SLAM methods

Ref.	Front end	Back end	Proposed method
[84]	BRISK and SURF	ASKF	Robust loop-closure approach
[85]	SIFT	I-EKF	Multi-session monocular underwater SLAM
[86]	FAST and IOF	GBA	Complex scene SLAM framework
[88]	NA	PGBA	Accurate, drift-free SLAM framework
[89]	SURF and RANSAC	FGBA	Ship hull surface reconstruction framework
[90]	NA	CANN	DolphinSLAM

BRISK, Binary Robust Invariant Scalable Keypoints; SURF, Speeded Up Robust Features; ASKF, Augmented State Kalman Filter; SIFT, Scale Invariant Feature Transform; I-EKF, Iterated Extension Kalman Filter; FAST, Features from Accelerated Segment Test; SLAM, Simultaneous Location and Mapping; IOF, Inverse Optical Flow; GBA, Global Bundle Adjustment; PGBA, Pose Graph Bundle Adjustment; RANSAC, Random Sample Consensus; FGBA, Factor Graph Bundle Adjustment; NA, None; CANN, Continuous Attractor Neural Network.

The Stereo-vision-based methods can obtain depth estimation according to the disparity map; theoretically, there is a smaller error compared to the monocular vision-based methods. Pi et al. [92] proposed a Stereo-SLAM framework with EKF. Nagappa et al. [93] proposed the single-cluster PHD SLAM, which was based on the Probability Hypothesis Density Filter (PHDF). Tank experiment results have shown a significant reduction in the localization error of the vehicle trajectory. Miao et al. [94] proposed the Unified Direct and Feature-Based Underwater Stereo Visual-Inertial Odometry (UniVIO), which had a robust front-end feature tracker with image preprocessing and robust data association capability. Aiming at the underwater feature-poor environments, Negre et al. [95] proposed a method with a novel loop-closure detection approach, which could effectively reduce the computational effort and system resource consumption without reducing the positioning accuracy. Billings et al. [71] proposed a visual feature-based method with robust mapping capability in the seafloor environments. The method successfully fuses the features from both a body stereo camera and a manipulator fisheye camera. Table 4 summarizes the underwater stereo visual SLAM methods.

**Table 4.** Summary of the reviewed underwater stereo visual SLAM methods

Ref.	Front end	Back end	Proposed method
[92]	SURF	EKF	A sparse SLAM framework
[93]	SURF	PHDF and EKF	Single-cluster PHD SLAM
[94]	GFTT	GBA	UniVIO
[95]	Feature cluster	PGBA	A framework for feature-poor environments
[71]	SIFT	Full BA	Hybrid visual SLAM

SURF, Speeded Up Robust Features; EKF, Extension Kalman Filter; PHDF, Probability Hypothesis Density Filter; GFTT, Good Features to Track; GBA, Global Bundle Adjustment; PGBA, Pose Graph Bundle Adjustment; BA, Bundle Adjustment.

The visual SLAM methods are not only easy to employ, but also efficient to reconstruct the environment map and achieve navigation. However, the unstructured underwater environments impose on the vision-based methods, especially the underwater SLAM, lots of great challenges:

• Because of sensor limitations, the underwater environment places special demands on the sensing equipment.

• Perceived difficulties, light degradation, and turbidity can affect the image quality.

• Compact computing resources and limited resources of the UVMS.

• Hard-to-meet robustness and real-time requirements.

#### Acoustic-based underwater navigation technology

Water serves as a good medium for sound transmission. Leveraging sound waves, the precise positioning and navigation of the underwater robots become feasible. Over the course of several decades of development, the acoustic-based methods have matured, emerging as the most commonly used approaches. Presently, there are mainly the following approaches: (a) baseline-based methods, (b) sonar-based methods, and (c) terrain-assisted methods.

The baseline-based approaches can be categorized into 3 groups based on the length of the receiving array: Ultra-Short Baseline (USBL), Short Baseline (SBL), and Long Baseline (LBL).

Wang et al. [96] designed a novel navigation system based on the Passive Inverted USBL (piUSBL). Localization and navigation can be achieved using only an inexpensive, low-power, and lightweight passive receiver and a periodically broadcasting beacon. Luo et al. [97] proposed an iUSBL-SLAM method based on Unscented Kalman Filter (UKF). Simulation results have shown that the proposed approach required only a few sparse beacons to estimate the vehicle’s position accurately. Nhat et al. [98] designed an active sonar navigation approach based on the USBL. Simulation results have shown that this method could efficiently increase the navigation accuracy in the 28 to 36 kHz frequency range. Zhang et al. [99] designed a smooth filtering algorithm for the LBL system, which could effectively reduce the positioning error of the system and improve the accuracy of underwater positioning. Aiming at the underwater environments with low signal-to-noise power ratios (SNRs), Lee et al. [100] proposed a USBL positioning method based on deep learning. The simulation results have shown that the proposed method has positioning performance that was up to 50 times better than the conventional methods. Table 5 presents the summary of the reviewed baseline-based methods. Following decades of research and development, baseline-based approaches have evolved into the pivotal and dependable solution for addressing positioning and navigation challenges, and had good performance in some specific applications.

**Table 5.** Summary of the reviewed baseline-based methods

Ref.	Approach	Main contribution
[96]	piUSBL	Accurate positioning with inexpensive, low-power, and lightweight equipment
[97]	iUSBL-SLAM and UKF	High positioning performance in sparse beacons environments
[134]	USBL time-delay estimation algorithm	High accuracy of TOF and TOA estimation in sound source navigation
[99]	LBL smooth optimizing algorithm	Long-range high accurate positioning performance
[100]	USBL and neural network	High positioning performance in low-SNR regions

piUSBL, Passive Inverted USBL; iUSBL, Inverted USBL; UKF, Unscented Kalman Filter; USBL, Ultra-Short Baseline; LBL, Long Baseline.

The sonar-based approaches are currently available for underwater 3D environmental mapping, localization, and navigation. Joe et al. [101] proposed a 3D mapping method using a Forward-Looking Sonar (FLS) and a Profile Sonar (PS). Experiment results demonstrated that the proposed method could improve elevation degradation and narrow-data issues. Wang et al. [102] proposed a 3D environment reconstruction framework using an acoustic camera, and solved the missing elevation angles of imaging sonars. The simulation results have shown that the framework could robustly and accurately reconstruct the dense 3D model of underwater targets and estimate the pose of the robot. Cheng et al. [103] proposed a Particle Filter (PF)-based underwater SLAM method with Multi-Beam Forward-Looking Sonar (MFLS). The experiment results have shown higher positioning accuracy compared with dead reckoning. Aiming at the small underwater vehicles with limited payloads and computing resources, Hwang [104] presented a UKF-SLAM method based on Range Sonar (RS). In 2022, Yang et al. [105] proposed the first mapless, low-cost, end-to-end underwater navigation method based on the Proximal Policy Optimization network (PPO) and Deep Reinforcement Learning (DRL). The proposed approach uses a fixed Single-Beam Echo Sounder (SBES) and a monocular camera to efficiently navigate the robot in unknown environments with obstacles. Table 6 presents a summary of the reviewed baseline-based methods. Overall, sonar-based methods have broad application potential in underwater environments, especially in deep seas and complex conditions. However, using sonar technology requires overcoming some challenges to ensure high accuracy and reliability. Therefore, in specific applications, sonar systems are often used with other sensors and technologies to compensate for their limitations.

**Table 6.** Summary of the reviewed sonar-based methods

Ref.	Equipment and approach	Main contribution
[101]	FLS and PS	Improve elevation degradation and narrow-data issues
[102]	Acoustic camera	Solve the missing elevation angles of imaging sonars
[103]	MFLS and PF	Good performance in both state estimation and suppressing divergence
[104]	RS and UKF	Low-power and real-time SLAM
[105]	PPO and DRL	The first mapless, low-cost, end-to-end underwater navigation method
[135]	FLS	Continuously accurate submarine pipeline tracking

FLS, Forward-Looking Sonar; PS, Profile Sonar; MFLS, Multi-Beam Forward-Looking Sonar; PF, Particle Filter; RS, Range Sonar; UKF, Unscented Kalman Filter; PPO, Proximal Policy Optimization; DRL, Deep Reinforcement Learning.

The TAN (Terrain-assist Navigation) technologies can be divided into prior-map-based methods [106] and mapless underwater terrain following navigation methods [107,108]. The former one has 3 primary components: reference navigation unit, terrain measuring unit, and terrain matching unit [73]. Conversely, the latter one mainly relies on a variety of underwater acoustic equipment to dynamically predict changes of the terrain and achieve autonomous follow-up navigation.

Song et al. [109] introduced an innovative underwater terrain matching method that combined digital topographic maps with real-time depth measurements from multi-beam sonars to efficiently improve matching accuracy. Ma et al. [73] proposed a 2-stage combined matching algorithm, and introduced the GA into the particle filter. Simulation and experiment results have shown that the proposed approach was superior to previous algorithms and was feasible in practical applications. Liu et al. [110] proposed a novel TAN system, based on fuzzy particle filter. Simulation results have demonstrated a high level of robustness and strong navigation ability under the ultra-low-resolution terrain map. Liao and Leng [111] proposed a method centered around the Intelligent Resampling Particle Filter. Aiming at the defects of particle degradation in the traditional particle filter algorithm, adjustments were made to the particle resampling strategy. Kurt et al. [112] provided a prior-map-free solution called Active TAN, and used an optimal, information-theoretic architecture and reinforcement learning (RL). The simulation results have verified the effectiveness of the architecture. These methods have a strong ability to adapt to the environment and can achieve long-term and large-scale navigation. However, due to the technical characteristics, TAN-based approaches will produce cumulative errors over time, and the equipment is costly with complex related technologies.

In brief, research into acoustic-based positioning and navigation methods was initiated during the 1950s. After decades of development, related technologies have gradually matured, leading to the productization, industrialization, and serialization of underwater acoustic positioning and navigation systems. Thanks to the good characteristics of sound waves in water, the acoustic-based approaches are expected to continue to receive continuous attention in the future.

#### Multisensory fusion navigation technology

With further research on the UVMS, demand for underwater operations continuously grows, and underwater missions are becoming considerably more complex. Simple sensor integrated positioning and navigation is no longer able to meet the requirements of these tasks. The growing performance gap has led to the development of multisensory fusion positioning and navigation technology, which encompass a framework that fuses information collected by multiple sensors. This technology can significantly enhance positioning accuracy and has strong robustness, and even achieve optimal real-time navigation.

Rahman et al. [113] proposed Svin2, which was a tightly coupled keyframe-based SLAM system using a sonar, a camera, and inertial and depth sensors. Information from various sensors is fused into a cost function *J*(*x*):

$$\begin{array}{c} J(x)=\sum_{i=1}^{2}\sum_{k=1}^{K}\sum_{j\in f(iK)},e_{r}^{i,j,k^{T}}P_{r}^{k}e_{r}^{i,j,k}+\sum_{k=1}^{K-1}e_{s}^{k^{T}}P_{s}^{k}e_{s}^{k}\\ \;+\sum_{k=1}^{K-1}e_{t}^{k^{T}}P_{t}^{k}e_{t}^{k}+\sum_{k=1}^{K-1}e_{u}^{k^{T}}P_{u}^{k}e_{u}^{k} \end{array}$$ (2)

where *i* denotes the camera index, left (*i* = 1) or right (*i* = 2) camera in a stereo camera system with landmark index *j* observed in the *k*th camera frame. $P_{r}^{k}$, $P_{s}^{k}$, $P_{t}^{k}$, and $P_{u}^{k}$ represent the information matrix of visual landmarks, IMU, sonar range, and depth measurement for the *k*th frame, respectively. Experiment results in low-visibility environments demonstrated that this method outperformed the existing state-of-the-art methods in both accuracy and robustness.

Aiming at complex environments, Wang et al. [114] proposed a tightly coupled navigation method using a Strap-down Inertial Navigation System (SINS), a Doppler Velocity Log (DVL), and a pressure sensor. Simulation and experiment results substantiate the method’s strong robustness. In comparison to loosely coupled methods, the navigation precision has been enhanced by 32.5%. Guo et al. [115] proposed a robust SINS/USBL navigation algorithm with Improved Statistical Similarity Measure Kalman Filter (ISSMKF), which effectively reduced the impact of acoustic outliers. Zhang et al. [116] proposed a SINS/DVL/SSS-based navigation method with factor graph optimization, and a side-scan sonar (SSS) was employed to minimize accumulation of position errors over time. In summary, underwater multisensory fusion navigation and positioning methods have advantages in improving the accuracy and robustness of underwater missions. However, it is necessary to address challenges such as complexity and cost.

#### Summary

The summary of these 3 positioning and navigation methods is as follows:

• The visual-based methods perform well in environment perception and target recognition. However, it is highly sensitive to illumination changes, occlusions, and adverse weather conditions, and performs poorly in complex environments.

• The underwater acoustic-based methods can achieve higher-precision positioning and navigation in wild underwater environment and also perform well in multiple target detection. However, the propagation of acoustic signals is easily affected by surrounding environments, and the processing and analysis of acoustic data are relatively complex.

• The multisensory fusion methods represent a novel navigation solution, offering heightened accuracy and robustness. By fusing data from various sensors, the limitations of their respective single technologies can be overcome. However, it is difficult to design a suitable fusion framework that can solve problems such as data synchronization and registration between sensors.

Underwater positioning and navigation technology is swiftly evolving, promising an exhilarating path ahead. As technology continues to progress, we anticipate that underwater positioning and navigation systems will enhance their precision and intelligence significantly. The next generation of positioning systems will leverage advanced sensor technology, deep learning algorithms, and bio-robot autonomous navigation technology [117], enabling a heightened perception and precise identification of the underwater environment.

### Autonomous operation control technology

The UVMS is a redundant kinematic system. Underwater autonomous operations mainly include robot motion control and coordinate control.

#### Motion control of the UVMS

As one of the key technologies for autonomous underwater operations, motion control can ensure precise positioning and trajectory tracking in unstructured underwater environments with unknown disturbance and obstacles. Motion control has remained a central focus of underwater robotics research for several decades, leading to the proposal of various control structures.

Bai [10] proposed an adaptive visual-servo-based underwater pipeline tracking control algorithm. Tracking experiments of various underwater pipelines were carried out in an indoor pool to verify the effectiveness of the proposed method. Cai [118] proposed an Improved Non-singular Terminal Sliding Mode Control (I-NTSMC) method with an Adaptive Differential Tracker (ADT), and Extended State Observer (ESO) was used to observe the disturbance. Time-varying jet disturbance was added during the underwater hovering experiments on Robcutt-II; the result verified the effectiveness of the proposed method. Vadapalli and Mahapatra [119] presented a robust state feedback optimal control method and designed the horizontal and vertical control algorithms, respectively. The simulation results have demonstrated the robust 3D-path tracking capability, even with unknown hydrodynamic coefficients. Heshmati-Alamdari et al. [120] proposed a dynamic robust Nonlinear Model Predictive Control (NMPC) approach, which could reasonably use ocean current to reduce energy consumption. Experiments have verified that this method could achieve autonomous navigation in the environment with sparse static obstacles.

The RL-based approach not only can effectively avoid the complicated modeling process, but also have strong robustness and strong adaptability to unknown environments. Tang [9] proposed a target tracking control method for undulatory fin-propelled underwater robots based on the Actor–Critic (AC) RL framework. The experiment results have indicated that even within highly perturbed environments, the robot remained capable of maintaining continuous tracking of the target. Aiming at hovering control of the UVMS, Ma et al. [121] proposed an RL approach based on the Soft Actor–Critic (SAC) framework, and a Neural Network (NN) was designed to fit nonlinear relationships involved in RL training. The experiment results of hovering control in an indoor pool verified the effectiveness of the proposed method. Table 7 shows the summary of the above motion control methods.

**Table 7.** Overview of the reviewed motion control methods

Ref.	Method	Model dependency	Task
[10]	Adaptive Visual-servo	Model-based	Pipeline tracking
[118]	I-NTSMC	Model-based	Autonomous door opening
[120]	NMPC	Model-based	Autonomous navigation
[119]	Optimal control	Model-based	3D-path tracking
[9]	Actor–critic RL	Model-free	Continuous target tracking
[121]	Soft actor–critic RL	Model-free	Stable hovering

I-NTSMC, Improved Non-singular Terminal Sliding Mode Control; NMPC, Nonlinear Model Predictive Control; RL, Reinforcement Learning.

At present, in the field of classic control, Sliding Mode Control (SMC), Model Predictive Control (MPC), and Intelligent Control have received extensive attention. For advanced control strategies, the focus is on combining or extending the classic methods, and adjusting and optimizing them for specific application scenarios [39]. In recent years, RL has played an increasingly important role, and simulation results have also verified the advantages of learning-based strategies. However, due to control stability challenges, there is a lack of actual pool experiments and real experiments in complex scenarios.

#### Vehicle-manipulator coordinated control

**Fig. 3.**
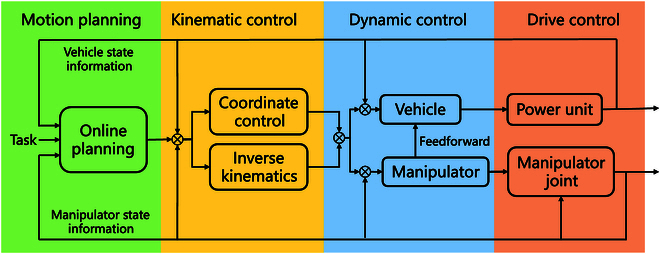
The vehicle-manipulator coordinated control framework [9].

In addition to motion control, vehicle-manipulator coordinated control is also an important part of autonomous operations. The UVMS is a kinematic-redundant system, so in order to achieve autonomous operations, it is necessary to coordinate the motion of the robot body and the manipulator. The vehicle-manipulator coordinated planning and control framework of the UVMS is shown in Fig. 3. The currently commonly used methods are as follows:

•Weighted minimum norm method: by introducing the weight matrix into the redundant system, and obtaining the minimum norm solution, and finally achieving motion planning. However, the conventional weighted minimum norm method exhibits constrained joint motion and cannot fully exert its kinematic performance within the allowed azimuth range [122].

•Gradient projection method: by introducing optimization constraints in the process of solving inverse kinematics, establishing optimization problems, and then optimizing performance indicators through gradient projection.

•Task priority method: by prioritizing multiple sub-tasks, solving each sub-task in turn, and ultimately achieving control of the system.

The weighted minimum norm method and the gradient projection method work well for a single task, but in the cases of multi-tasks, conflicts between tasks may occur. The task priority method can resolve these conflicts. Based on the task-priority method, Tang et al. [123] provided a redundancy resolution based on acceleration level. Saebo et al. [124] proposed a robust impedance-based task-priority control method, and simulation results showed effectiveness and robustness of proposed approaches. Wen et al. [125] presented a novel visual-served task-priority method, which could realize visual-based remote motion planning. Due to the outstanding performance, the task priority method has been applied for many underwater autonomous operation tasks in recent years. The applications of the task-priority method is shown in Table 8.

**Table 8.** Application examples of task-priority methods

Application platform	Year	Task	Experiment or simulation
I-AUV [136]	2013	Objects recovering	Pool and marine environments
GIRONA 500 [135]	2014	Salvage of black boxes	Marine environments
GIRONA 500 [136]	2015	Valve turning	Pool
H2020 DexRov [43]	2017	Pipe welding	Gazebo (simulation)
I-AUV [137]	2017	Connector plug/unplug	Pool
H2020 ROBUST [138]	2019	Seabed mineral sampling	Pool and marine environments
TWINBOT [139]	2021	Cooperative transportation	Pool

In recent years, alongside the above model-based control methods, some model-free methods have been achieved with the support of NN and RL. The EU proposed a parametric Learning by Demonstration strategy (p-LbD) in the PANDORA project [126]. The underwater free-floating valve turning experiment results have shown the feasibility of the p-LbD algorithm for executing autonomous intervention tasks.

#### Summary

While significant advancements have been achieved in the underwater operation control, it is important to note that the majority of these methods have been primarily validated through numerical simulations. Some relatively simple tasks such as plugging/unplugging, docking, and grasping were carried out by pool experiments. There is still a lack of platform verification in the actual marine environment.

## Discussion

In this work, some application examples of the UVMS are discussed, which show the possible application prospects in underwater environments. Current and future developments and limitations of the UVMS are discussed below, which will provide insights for further research.

Underwater robots are the result of interdisciplinary fusion. The future development of the UVMS will integrate various advanced technologies such as artificial intelligence, detection and recognition, information fusion, intelligent control, and system integration [127]. These technologies will enable robots to liberate themselves from real-time human intervention and be able to autonomously make decisions and achieve completely autonomous control. Strong learning abilities will allow robots to adapt to changes of the environment through learning, and can be capable of independently carrying out various tasks in complex marine environments. Future research will primarily encompass the following areas:

• Structure Design Optimization. The future development direction of the UVMS will be small size, long endurance, and task reconfiguration. Motion maneuverability and operation stability can be imroved by optimizing the shape design of the UVMS, selecting suitable new materials, optimizing the space layout among thrusters, and developing energy supply systems with high energy density and bionic-propulsion technology.

• Manipulator Structure Design Optimization. The optimization of manipulator can greatly improve the efficiency of underwater operations, especially in unstructured environments. Some bionic designs can be added to the UVMS system, such as bioinspired soft robotic manipulators that adapted to unstructured environments [128] and bionic bimanual manipulators [129].

• Environment Aware Ability. Multisensory fusion can enhance the aware capabilities of the UVMS in large-scale outdoor unstructured environments and improve the application ability of underwater operation robots in complex underwater scenes. With the help of underwater computer vision, deep learning, and SLAM technologies, in-depth perception of the underwater environment can be achieved, including multi-type target detection, target positioning in complex environments, large-scale map construction, etc.

• Autonomous Operation Ability. Further in-depth research is needed for autonomous control methods in complex scenarios, 3D spaces, time-varying turbulence, and continuous tasks. It is also necessary to enhance the motion planning ability of the manipulator and the ability to interact with the environment.

• Good Learning Ability. Aiming at the underwater operation environment with disturbances, combining with control strategies such as RL, transfer learning, deep learning, and biological neural networks [130] can realize autonomous search and positioning of operating targets in unstructured environments, autonomous task planning, coordinated control of the body and manipulators, and even multi-underwater robot cluster operations and multi-platform collaborative operations.

## Conclusion

This paper summarizes the development status of the UVMS and some key technologies. First, this paper presents an overview of recent research and trends in the UVMS and introduces some typical types in detail. Most of the classic control methods rely on the dynamic and hydrodynamic model. This article provides a brief overview of dynamic modeling methods including the Newton–Euler method, the Quasi-Lagrange method, the Kane method, the Finite Element Analysis method, the Data-driven method, and the Hybrid method. In addition, hydrodynamic modeling methods are divided into 4 main categories: approaches based on the EF, RT, NSA, and MPI. The advantages and disadvantages of various modeling methods are analyzed. The purpose is to provide ideas for researchers to solve relevant modeling problems. The operation environment of the UVMS is complex and requires reliable positioning and navigation. This article tries to make a brief review of 3 different positioning and navigation technologies. Vision-based approaches are easy to deploy and use. However, it is difficult to meet the robustness requirement, which can easily be affected by surrounding environments. Underwater acoustic-based methods are commonly used. Baseline-based approaches can provide long-range, long-time accurate positioning and navigation, but there are difficulties in deployment and calibration; sonar-based methods work well underwater, but there are difficulties in data processing and reliability; TAN methods have strong environmental adaptability, but it is expensive and complex. The multisensory fusion methods can combine the advantages of various sensors and have better performance. Motion control can ensure precise positioning and trajectory tracking in unstructured underwater environments with unknown disturbance. This paper gives a brief overview of some typical classic control methods and learning-based control approaches. When operating underwater, the manipulator will affect the body, so the vehicle-manipulator coordinate control needs to be considered. This paper summarizes the current commonly used coordinate control methods and discusses their respective principles and some application examples. Finally, this paper discusses the future direction of the UVMS and corresponding key technologies.

Humanity’s enduring fascination with the ocean persists, and while technological limitations have constrained our ability to explore its depths, progress remains unstoppable. As a pivotal force driving future ocean exploration, the UVMS will undoubtedly evolve alongside technological advancements. The ultimate aspiration of human ocean exploration and development is destined to be realized.

## Data Availability

The data used to support the findings of this study are available from the corresponding author upon request.
